# MyD88 contribution to ocular surface homeostasis

**DOI:** 10.1371/journal.pone.0182153

**Published:** 2017-08-10

**Authors:** Rose Y. Reins, Justin Courson, Carolina Lema, Rachel L. Redfern

**Affiliations:** The Ocular Surface Institute, College of Optometry, University of Houston, Houston, Texas, United States of America; Wayne State University, UNITED STATES

## Abstract

The cornea must maintain homeostasis, enabling rapid response to injury and microbial insult, to protect the eye from insult and infection. Toll-like receptors (TLRs) are critical to this innate immune response through the recognition and response to pathogens. Myeloid differentiation primary response (MyD88) is a key signaling molecule necessary for Toll-like receptor (TLR) and interleukin-1 receptor (IL-1R)-mediated immune defense and has been shown to be necessary for corneal defense during infection. Here, we examined the intrinsic role of TLR signaling in ocular surface tissues by determining baseline levels of inflammatory mediators, the response to mechanical stimuli, and corneal infection in MyD88-deficient mice (MyD88^-/-^). In addition, cytokine, chemokine, and matrix metalloproteinase (MMP) expression was determined in ocular surface cells exposed to a panel of TLR agonists. Compared to wild-type (WT) animals, MyD88^-/-^ mice expressed lower MMP-9 levels in the cornea and conjunctiva. Corneal IL-1α, TNFα, and conjunctival IL-1α, IL-2, IL-6, and IL-9 levels were also significantly reduced. Additionally, CXCL1 and RANTES expression was lower in both MyD88^-/-^ tissues compared to WT and IL-1R^-/-^ mice. Interestingly, MyD88^-/-^ mice had lower corneal sensitivities (1.01±0.31 gm/mm^2^) than both WT (0.59±0.16 gm/mm^2^) and IL-1R^-/-^ (0.52±0.08 gm/mm^2^). Following *Pseudomonas aeruginosa* challenge, MyD88^-/-^ mice had better clinical scores (0.5±0.0) compared to IL-1R^-/-^ (1.5±0.6) and WT (2.3±0.3) animals, but had significantly more corneal bacterial isolates. However, no signs of infection were detected in inoculated uninjured corneas from either MyD88 or IL-1R-deficient mice. This work furthers our understanding of the importance of TLR signaling in corneal defense and immune homeostasis, showing that a lack of MyD88 may compromise the baseline innate response to insult.

## Introduction

The corneal and conjunctival epithelium form the anterior covering of the eye and provide the first line of defense against damage and injury, as well as protection against invading pathogens. One of the primary functions of these tissues is to provide a barrier, preventing infection and physical damage to the rest of the eye. Therefore, innate immune defense mechanisms and maintaining tissue homeostasis are vitally important to the ocular surface. Toll-like receptors (TLRs) are pattern-recognition receptors that mediate innate immunity through recognition of pathogen-associated molecular patterns (PAMPs) on microbial ligands and endogenous damage-associated molecular patterns (DAMPs) [1–3]. Activation of TLRs results in the production of cytokines, chemokines, and other mediators which signal to neighboring cells, recruit immune cells to the site of activation, trigger extracellular matrix changes, and stimulate an adaptive immune response against infection. TLRs also stimulate antimicrobial peptide production, which mediate pathogen killing, modulate immune signals, and play a role in wound healing [4–6].

Thirteen mammalian TLRs have been identified to date, which recognize conserved moieties on proteins, lipids, and nucleic acids. Upon ligand binding, TLR engagement leads to a cascade of intercellular signaling events that culminate in nuclear factor kappa B (NF-κB) and interferon regulatory factor (IRF) transcription factor activation and the production of pro-inflammatory cytokines, chemokines, and type-1 interferons [3,7,8]. Initiation of these signaling pathways is mediated by adaptor proteins that bind the cytoplasmic domain of TLRs, through a Toll/IL-1 receptor (TIR) domain. Myeloid differentiation primary response gene 88 (MyD88) is the most well characterized adaptor and is the primary pathway for TLR-mediated responses [2,9]. In addition to TLRs, the interleukin (IL)-1 receptor (IL-1R) also utilizes MyD88 for signal transduction.

TLRs have been shown to be expressed at the ocular surface, playing an important role in defending against infection and in initiating inflammation #[9–12]. TLR2 and MyD88 have been implicated in *Staphylococcus aureus* virulence [13,14], while TLRs 4, 5 and MyD88 are required for effective clearance of *Pseudomonas aeruginosa* (PA) [15–18]. TLRs/MyD88 signaling also mediate the corneal response to viral, fungal, *Acanthamoeba*, and nematode infections [19–22]. The TLR response is necessary and beneficial to the cornea, as mice deficient in TLRs are more susceptible to microbial keratitis [9,13,15,16,18].

TLR activation leads to IL-1, IL-6, and tumor necrosis factor alpha (TNFα) production at the ocular surface, which contribute to the initiation and propagation of inflammatory or danger signals. Chemokines such as C-X-C motif chemokine ligand 1 and 2 (CXCL1, 2) are also important mediators of TLR-mediated corneal defense, attracting leukocytes to the area of TLR activation. In addition, TLRs augment the production of matrix metalloproteinases (MMPs), which are involved in normal tissue remodeling and extracellular matrix turnover, as well as matrix degradation, cytokine activation, and cell proliferation during wound healing and inflammation [23]. While levels of these inflammatory mediators must be kept in check to prevent tissue damage and chronic inflammation, which compromise corneal transparency, they are known to be expressed in normal, non-diseased tissue, and may contribute to the rapid response necessary in protecting against infection and injury [24–26]. Therefore, it is relevant to examine how MyD88 and TLR signaling contribute to baseline levels of inflammatory protein expression.

TLRs have also been implicated in nerve function and induction of chronic pain [27–31]. Spinal TLRs and MyD88 signaling have been shown to initiate neuropathic allodynia following spinal nerve injury [30]. TLR2 and TLR4 deficient mice exhibit reduced pain behavior and affected nerve recovery following injury [29,31]. In addition, TLR2 and TLR4 agonists stimulated the production of nerve growth factor in human intervertebral discs [28]. However, TLR involvement in corneal nerve function has not been characterized. In the current study, we examined the intrinsic role of TLR signaling in ocular surface tissues by determining baseline levels of inflammatory mediators, the response to mechanical stimuli, and corneal infection in MyD88 and IL-1R-deficient mice.

## Materials and methods

### Human cell culture and TLR agonist treatment

For primary cell isolation, human cadaveric donor corneas and conjunctival tissue were obtained from Saving Sight Eye Bank (St. Louis, MO) within one week of donor death. Primary human corneal epithelial cells (HCEC) were isolated by enzymatic digestion (Dispase II, Roche Diagnostics, Indianapolis, IN) and cultured in EpiLife medium with defined growth supplement (Invitrogen, Grand Island, NY), 60μM calcium chloride, and penicillin-streptomycin (5000U/mL; 5000μg/mL), as previously described [32]. Primary human conjunctival epithelial cells (HConjEpi) were isolated from conjunctival donor tissue following digestion with 0.05% Trypsin-EDTA (Sigma-Aldrich, St. Louis, MO) and maintained in KGM-2 medium (Lonza, Allendale, NJ) [33]. Human telomerase-immortalized corneal epithelial cells (hTCEpi) were cultured as previously described [34]. Prior to treatment, all cells were grown to approximately 75% confluency before use in experiments. Cells were stimulated with TLR agonists [1μg/ml Pam3CSK4 (TLR1/2), FSL1 (TLR6/2), LPS *E*.*coli K12* (TLR4), Flagellin *Salmonella typhimurium* (TLR5), Imiquimod (TLR7), ssRNA (TLR8), 10^8^ cells/ml heat-killed *Listeria monocytogenes* (HKLM, TLR2), or 2μM ODN2006 (TLR9); Invivogen, San Diego, CA] or IL-1β 10ng/ml) for 24 hours.

### MMP and cytokine protein analyses

Following treatment with TLR agonists for 24 hours, human MMPs (MMP-1, MMP-2, MMP-7, MMP-9 and MMP-10) and cytokines (IL-1β, IL-2, IL-4, IL-5, IL-6, IL-7, IL-8, IL-10, IL-12, IL-13, GM-CSF, IFNγ, and TNFα) were detected in cell culture supernatants with MILLIPLEX MAP Human Magnetic Bead Panels (EMD Millipore, San Diego, CA), per manufacturer’s instructions. IL-6 and IL-8 expression was confirmed in primary HCEC supernatants by ELISA (Biolegend, San Diego, CA). For mouse corneal epithelial and conjunctival samples (6 mice per sample, n = 3 experiments), pooled tissue was homogenized in 0.2% Triton X-100 containing a protease inhibitor cocktail (Roche, Nutley, NJ). MMPs (MMP-2, MMP-3, MMP-8, proMMP-9, MMP-12) and cytokines/chemokines (IL-1β, IL-2, IL-4, IL-5, IL-6, IL-7, IL-9, IL-10, IL-12, IL-13, IL-15, IL-17, IP-10, MKC, MCP-1, MIP-1α, MIP-1β, MIP-2, G-CSF, GM-CSF, IFNγ, TNFα, CXCL1, and RANTES) were quantitated using MILLIPLEX MAP Mouse MMP Magnetic Bead Panel 3 and Cytokine/Chemokine Panel- Immunology Multiplex Assays. Total protein concentrations were determined in supernatants and tissue samples by Direct Detect Assay Kit (EMD Millipore) and 10μg of total protein was loaded per well in duplicate.

### Mice

Eight to twelve week old C57BL/6 (wild-type strain, WT), MyD88-deficient (MyD88^-/-^), and IL-1R-deficient (IL-1R^-/-^) mice were purchased from The Jackson Laboratory (Bar Harbor, ME). Animal experiments were approved by the Institutional Animal Care and Use Committee at the University of Houston and adhered to the standards of the Association for Research in Vision and Ophthalmology Statement for the use of animals in ophthalmic and visual research.

### RNA isolation and quantitative real-time PCR

For RNA isolation, mouse corneal epithelial cells were removed by scraping and the conjunctival tissue was surgically dissected, as previously reported [10]. Three mice were pooled from the same genotype for each sample (n = 3 independent experiments; 15 mice per genotype). Total RNA was extracted from each sample using RNeasy kits with DNase I treatment (Qiagen, Valencia, CA) and reverse transcribed with an AffinityScript cDNA synthesis kit (Agilent Technologies, Santa Clara, CA). Real-time PCR was performed using intron-spanning primers and Brilliant II SYBR Green QPCR master mix (Agilent Technologies) for *CXCL1* (forward: TGCACCCAAACCGAAGTC; reverse: GTCAGAAGCCAGCGTTCACC), *IL1A* (forward: CAGGGCAGAGAGGGAGTCAAC; reverse: CAGGAACTTTGGCCATCTTGAT), *TNFA* (forward: ACTGAACTTCGGGGTGATCG; reverse: TGATCTGAGTGTGAGGGTCTGG), and *MMP9* (forward: CAGCCAACTATGACCAGGAT; reverse: CTGCCACCAGGAACAGG). All samples were normalized to the housekeeping gene, *RPII* (forward: CTACACCACCTACAGCCTCCAG; reverse: TTCAGATGAGGTCCATGAGGAT), for relative quantity determination using the ΔΔCT method.

### Corneal sensitivity

Mice corneal sensitivity was measured using a Cochet-Bonnet esthesiometer, as previously reported [35]. Briefly, the esthesiometer monofilament was applied to the central cornea and the length was varied in 0.5cm increments from 6.0 to 0.5cm until the threshold sensitivity was reached, observed by a blink. Measurements were taken by the same examiner in each experiment and a second examiner recorded animal blinks. At each length, the monofilament was applied 4 times and threshold sensitivity was reached when the animal responded with a blink in more than 50% of the stimulations.

### Experimental bacterial keratitis

To study corneal infection *in vivo*, C57BL/6 wild type (WT), IL-1R^-/-^, and MyD88^-/-^ were infected with GFP-PA01, a PA strain expressing green-fluorescent protein and carbenicillin resistance gene (generously given by Dr. Alice Prince, Columbia University, NY), as previously described [36]. Briefly, mice were anesthetized by intraperitoneal injection of ketamine (60mg/kg)/xylazine (6mg/kg) (Vedco, Inc., St. Joseph, MO). With the aid of a dissecting microscope, three parallel, epithelial, 1-mm scratches were made in the right corneas of mice using a sterile 27-gauge needle, or corneas were left unscratched. In one set of animals, 5μl of bacterial suspension, 1.0 × 10^6^ colony forming units (CFU) GFP-PA01 in PBS, was pipetted onto the surface of scratched corneas (right eyes), as well as unwounded corneas (left eyes), which served as controls. A second set of animals received no scratch in either eye, with right eyes inoculated with GFP-PA01 and left eyes received 5μl of PBS, vehicle control. After 6 or 24 hours, eyes were imaged by slit lamp microscopy to grade the clinical progression of the infection. Severity of corneal damage was determined based on the following grading scale of 0–4: 0, clear or slight opacity, partially covering the pupil; +1, slight opacity, fully covering the cornea; +2, dense opacity, partially or fully covering the pupil; +3, dense opacity fully covering the cornea; and +4, corneal perforation or phthisis [37].

All mice were euthanized by carbon dioxide asphyxiation followed by cervical dislocation following imaging, at 24 hours post-infection. To obtain viable bacterial counts, corneas were harvested, homogenized in PBS, serially diluted, and 10μl of the homogenate was plated in duplicate onto Difco Luria-Bertani (BD Biosciences, San Jose, CA) nutrient agar plates containing carbenicillin (300μg/ml). Plates were incubated for 16 hours at 37°C to allow bacterial growth and the number of CFU was counted. To visualize GFP-expressing PA, corneal whole mounts were prepared, as previously reported [38]. Briefly, whole eyes were removed, fixed with 2% paraformaldehyde, and corneas dissected. Corneas were then flattened with radial cuts and mounted onto slides for imaging with a DeltaVision Core microscope (Applied Precision, Issaquah, WA).

### Statistical analyses

Statistical analyses were performed using ANOVA (one-way or two-way dependent on the number of independent variables tested), with Bonferroni’s test for multiple comparisons. All data are representative of a minimum of three independent experiments with p ≤ 0.05 considered statistically significant. Statistical tests were performed with GraphPad Prism 6.0 software (GraphPad Software Incorporation, San Diego, CA).

## Results

### TLR agonists increased MMP and cytokine expression in various ocular surface cells

**Fig 1:**
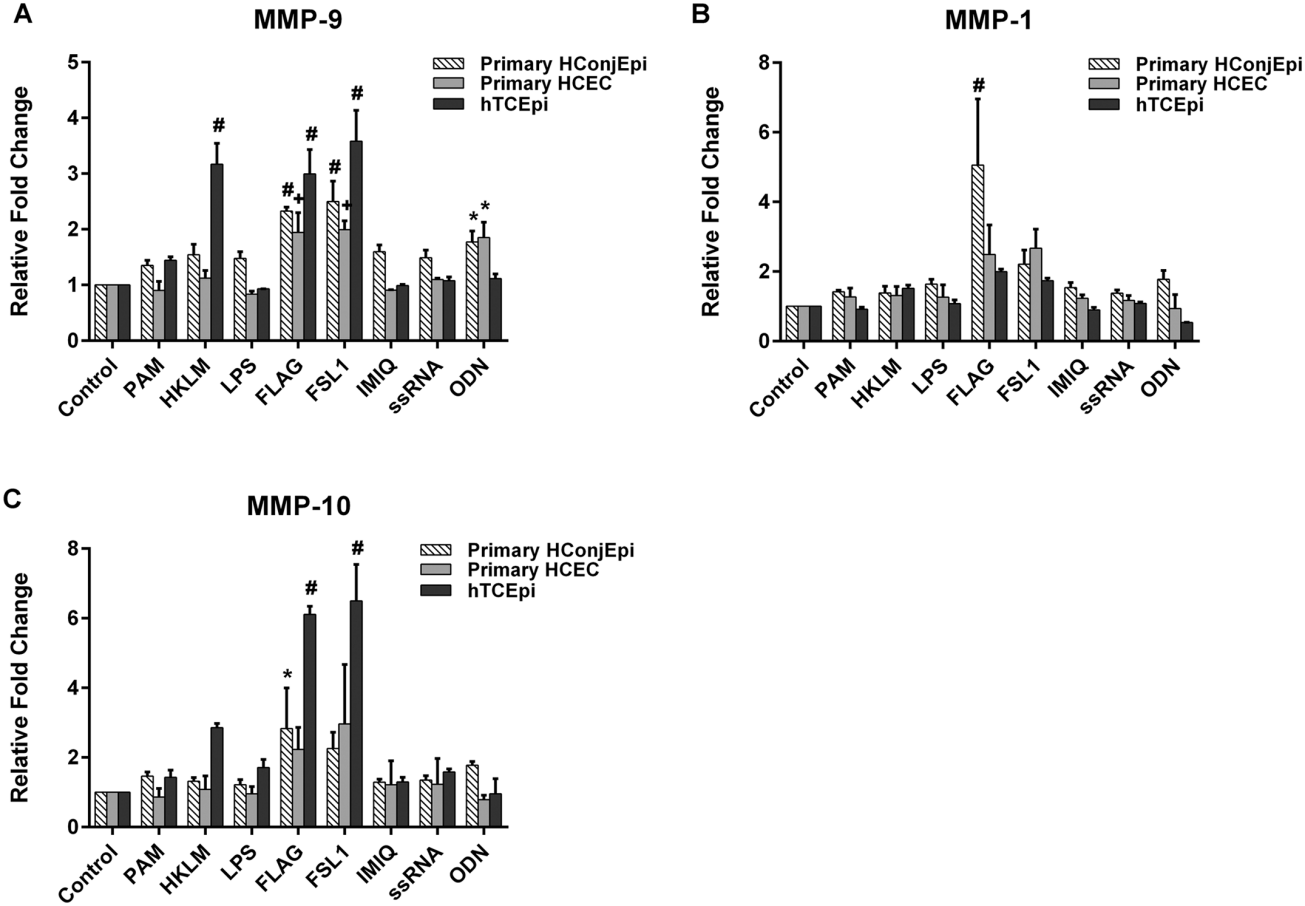
TLR activation increases levels of MMPs in human corneal and conjunctival cells. Primary HCEC, hTCEpi, and primary HConjEpi were left untreated (control), or were treated with TLR agonists [TLR2/1 (PAM), TLR2 (HKLM), TLR4 (LPS), TLR5 (FLAG), TLR6/2 (FLS-1), TLR7 (IMQ), TLR8 (ssRNA), or TLR9 (ODN)] for 24 hours. Pro-MMP-9 (A), MMP-1 (B), and MMP-10 (C) expression was determined in cell supernatants by Luminex bead assay. Graphs represent mean ±SEM of 3 independent experiments (n = 3). Analysis was performed by ANOVA with Bonferroni’s test for multiple comparisons. p < *0.05, +0.01, #0.0001.

**Fig 2:**
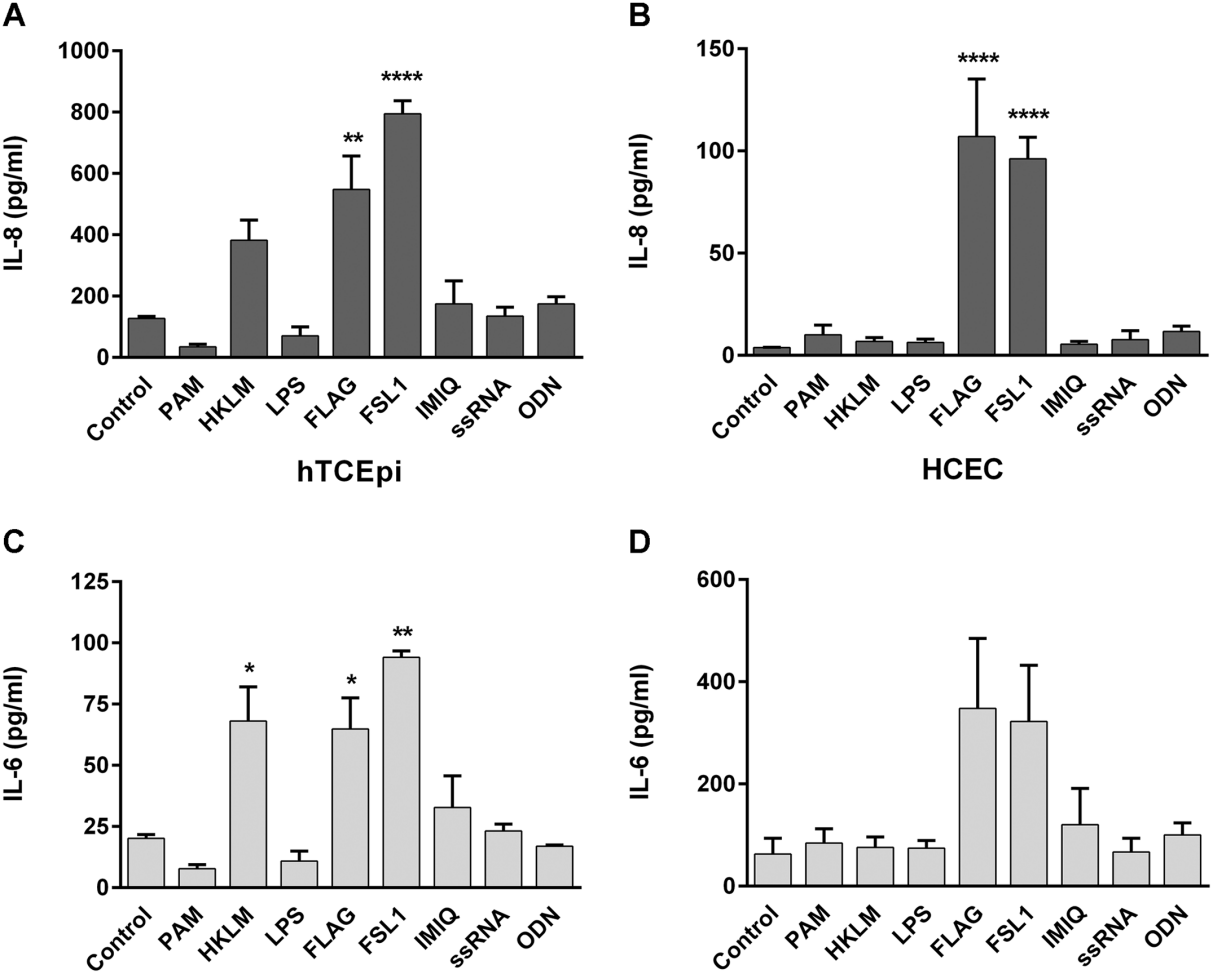
TLR agonist treatment induces cytokine expression in human corneal cells. hTCEpi and primary HCEC were treated with TLR agonists [TLR2/1 (PAM), TLR2 (HKLM), TLR4 (LPS), TLR5 (FLAG), TLR6/2 (FLS-1), TLR7 (IMQ), TLR8 (ssRNA), or TLR9 (ODN)] for 24 hours and IL-8 (A, B) and IL-6 (C, D) expression was determined in cell supernatants by Luminex bead assay (hTCEpi) or ELISA (HCEC). Graphs represent mean ±SEM of 3 independent experiments (n = 3). Analysis was performed by ANOVA with Bonferroni’s test for multiple comparisons. p < *0.05, **0.01, ****0.0001.

One of the major functions of the ocular surface is defending the rest of the eye from damage and infection. *In vitro* and *in vivo* studies demonstrate the importance of TLR activation in the initiation and propagation of inflammation, through production of immune-regulating mediators that aid in ocular surface innate defense. As our aim was to evaluate the expression of these mediators in ocular surface tissues and their dependence on TLR signaling, we first sought to analyze the effect of TLR signaling *in vitro* on MMP (Fig 1) and cytokine (Fig 2) expression in various corneal and conjunctival cells. Primary corneal epithelial cells (HCEC), primary conjunctival epithelial cells (HConjEpi), and an immortalized corneal epithelial cell line (hTCEpi) were treated for 24 hours with TLR agonists that mediate their effects through the signaling adaptor protein, MyD88, and were specific for TLR2 (Pam3CSK4, FSL1), TLR4 (LPS), TLR5 (Flagellin, FLAG), TLR7 (Imiquimod, IMIQ), TLR8 (ssRNA), and TLR9 (ODN2006, ODN) (TLR9). For MMP-9 expression, flagellin and FSL1increased protein supernatant levels across all cell types (1.9 to 3.0-fold, flagellin; 2.0 to 3.6-fold, FSL1), while the TLR2/1 agonist, HKLM, and TLR9 agonist ODN exerted variable effects between cells (Fig 1A). Similarly, flagellin and FSL1 treatment increased MMP-10 expression above untreated control levels in hTCEpi (Fig 1C). Interestingly, although relative fold change in MMP-10 was greatest with agonist treatment in hTCEpi, baseline expression was lowest in hTCEpi, with untreated hConjEpi expressing more than 10-fold higher MMP-10 concentrations (hConjEPI, 4141±81pg/ml vs. HCEC, 1965±64 pg/ml and hTCEpi, 157±9 pg/ml). TLR agonist treatment did not elicit a significant increase in MMP-1 expression, with the exception of flagellin, which increased relative amounts of hConjEpi MMP-1 by 5-fold (Fig 1B).

In addition to MMPs, TLR signaling also induces the expression of pro-inflammatory cytokines, which signal to neighboring cells in response to infection and inflammation. To determine the corneal cytokine response to stimulation, hTCEpi and primary HCEC were treated with a panel of TLR agonists, as above, for 24 hours. Similar to MMP expression, flagellin and FSL1 induced significant increases in both IL-8 (hTCEpi: flagellin-treated 548±110 and FSL1-treated 794±43 vs. control 127±6 pg/ml; HCEC: flagellin-treated 107±28 and FSL1-treated 96±11 vs. control 4.0±0.2 pg/ml) and IL-6 expression (hTCEpi: flagellin-treated 65±13 and FSL1-treated 94±3 vs. control 20±2 pg/ml; HCEC: flagellin-treated 348±137 and FSL1-treated 322±110 vs. control 127.42±6.32 pg/ml). In addition, TNFα expression in hTCEpi cell supernatants increased with flagellin (7.4±0.9 pg/ml) and FSL1 (7.86±0.6 pg/ml), compared to unstimulated levels (media, 1.3±0.2 pg/ml) (data not shown). These results confirm that TLR activation, through agonist treatment that signal through MyD88, leads to the upregulation of cytokines and MMPs in ocular surface cells, which are necessary in the response to infection and injury.

### MyD88^-/-^ mice have decreased expression of MMPs, pro-inflammatory cytokines, and chemokines at the ocular surface

As TLR stimulation led to enhanced expression of inflammatory mediators, we hypothesized that disruption of the common signaling pathway, through the adaptor protein MyD88, would lead to lower basal levels of these mediators *in vivo*, which are important in defending the ocular surface from damage. Therefore, we sought to evaluate the effect of MyD88 deficiency on the normal, unchallenged expression of these inflammatory mediators. We also were interested in determining the relative contributions of TLR or IL-1β signaling in the expression of these molecules, and therefore also examined IL-1R deficiency.

**Fig 3:**
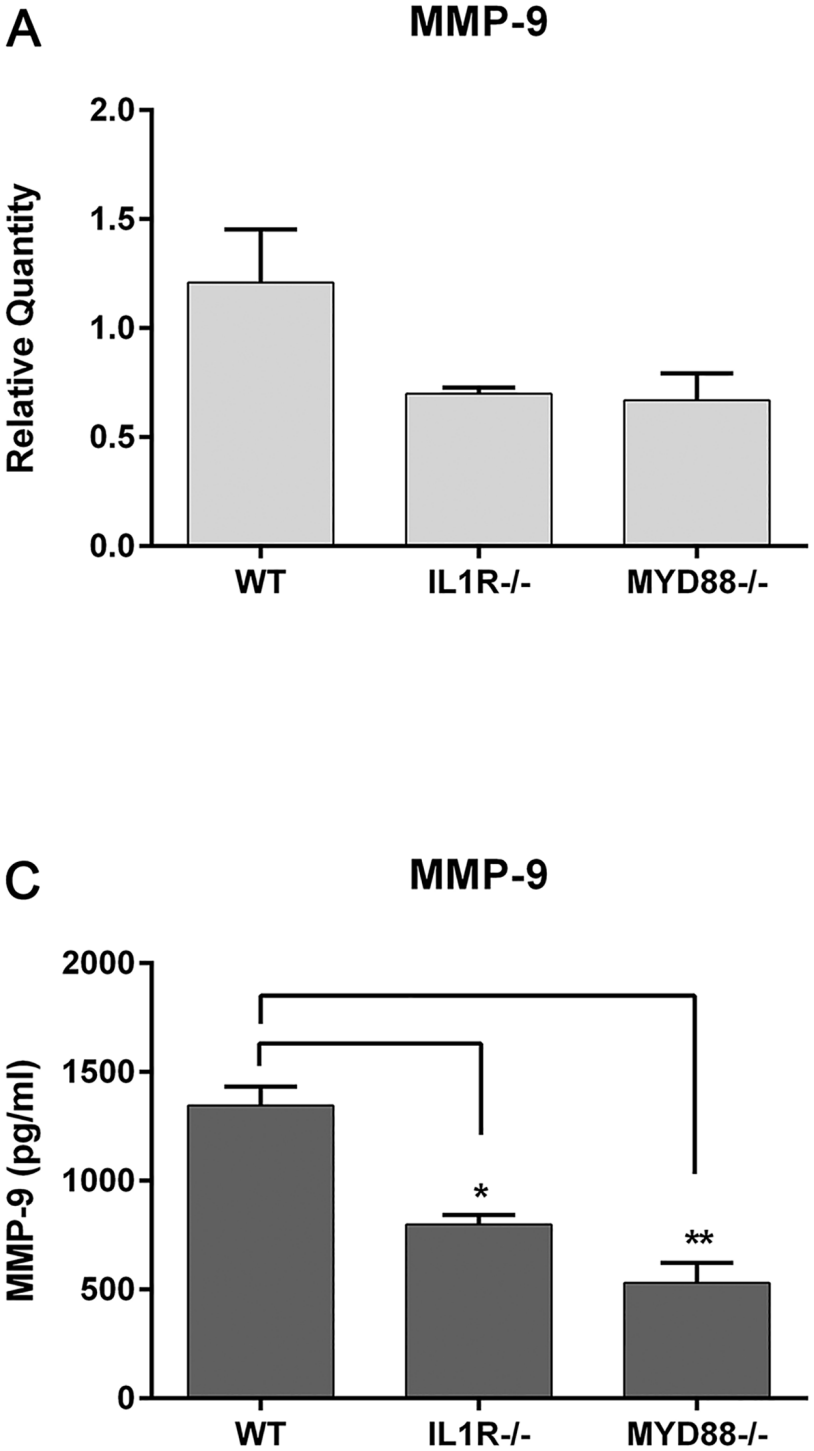
MyD88-deficient mice have lower levels of MMP expression in the cornea and conjunctiva. (A) MMP-9 expression was determined in untreated corneal lysates by RT-PCR. Graph represent mean ±SEM of 3 independent experiments with each sample pooled from 4 mice. (B) MMP-9 protein expression was quantitated in conjunctival homogenates by Luminex multiplex assay. Samples represent 10 pooled corneas per genotype. Graph represent mean ±SEM of 3 independent experiments (n = 3). Analysis was performed by ANOVA with Bonferroni’s test for multiple comparisons. p < *0.05, **0.01.

Corneal cells and conjunctival tissue were removed from unchallenged WT (C57), IL-1R^-/-^, and MyD88^-/-^ mice and MMP-9 gene or protein expression evaluated. Both knockout strains had lower relative MMP-9 gene expression in the cornea compared to WTs (WT, 1.2±0.2; IL-1R^-/-^, 0.7±0.03; MyD88^-/-^, 0.7±0.07 relative fold changes) (Fig 3A). MMP-9 protein expression was also significantly decreased in deficient mice, with the lowest levels in the MyD88^-/-^ conjunctival tissue (530.9±92.0 pg/ml compared to WT, 1345.0±87.0 pg/ml) (Fig 3B). MyD88^-/-^ and IL-1R^-/-^ mice also had lower expression of MMP-8 in the conjunctiva.

**Fig 4:**
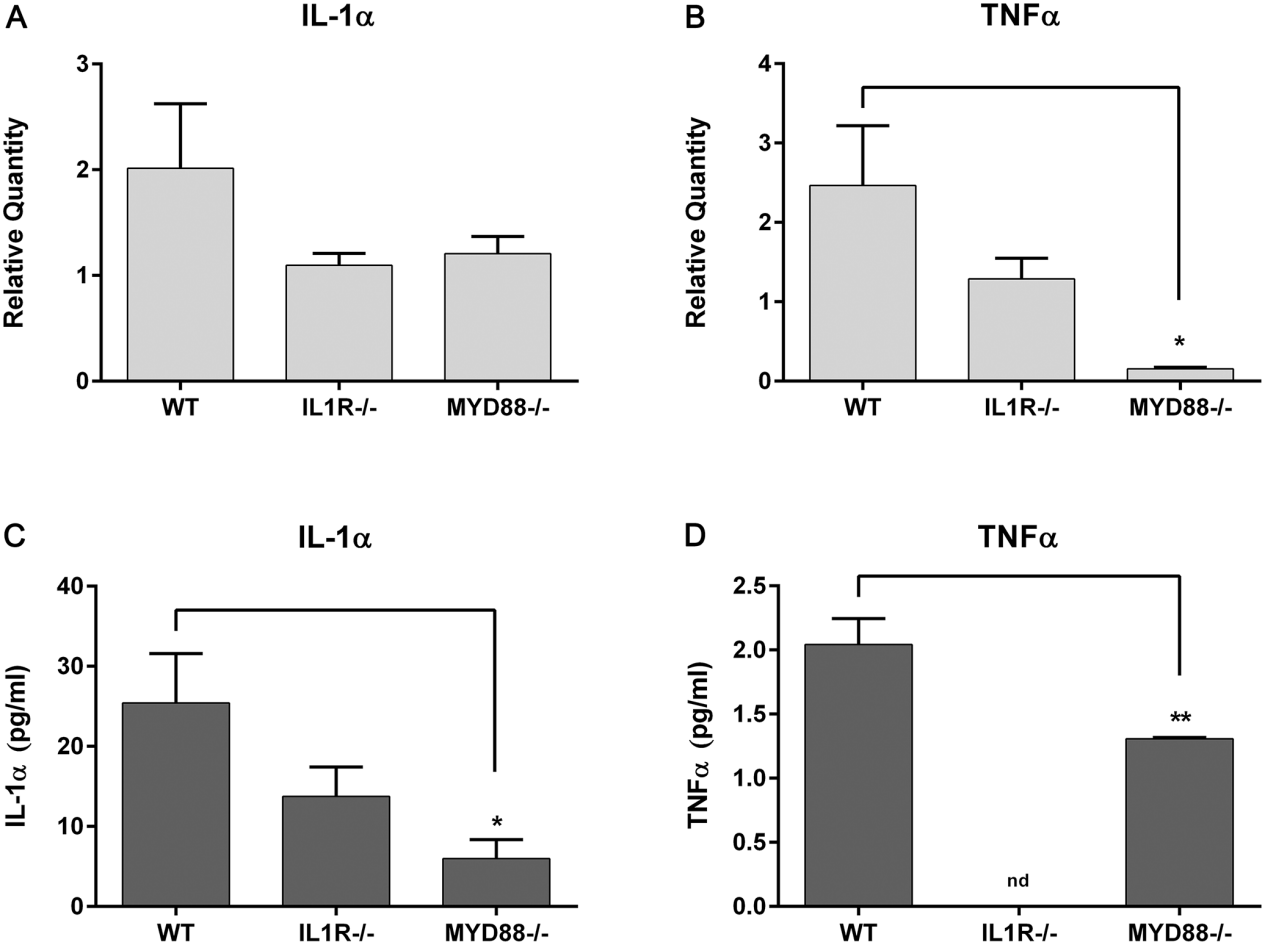
Lack of TLR signaling (MyD88^-/-^) results in decreased levels of cytokines in the cornea. IL-1α (A, C) and TNFα (B, D) gene expression was determined in untreated corneal lysates by RT-PCR (top) and protein expression quantitated in corneal homogenates by Luminex multiplex assay (bottom), in wild-type (WT), IL-1R^-/-^, and MyD88^-/-^ mice. Graphs represent mean ± SEM of 3 independent experiments with each sample pooled from 5 mice. Analysis was performed by ANOVA with Bonferroni’s test for multiple comparisons, with comparison to WT samples. p<*0.05, **0.01, nd = not determined.

**Fig 5:**
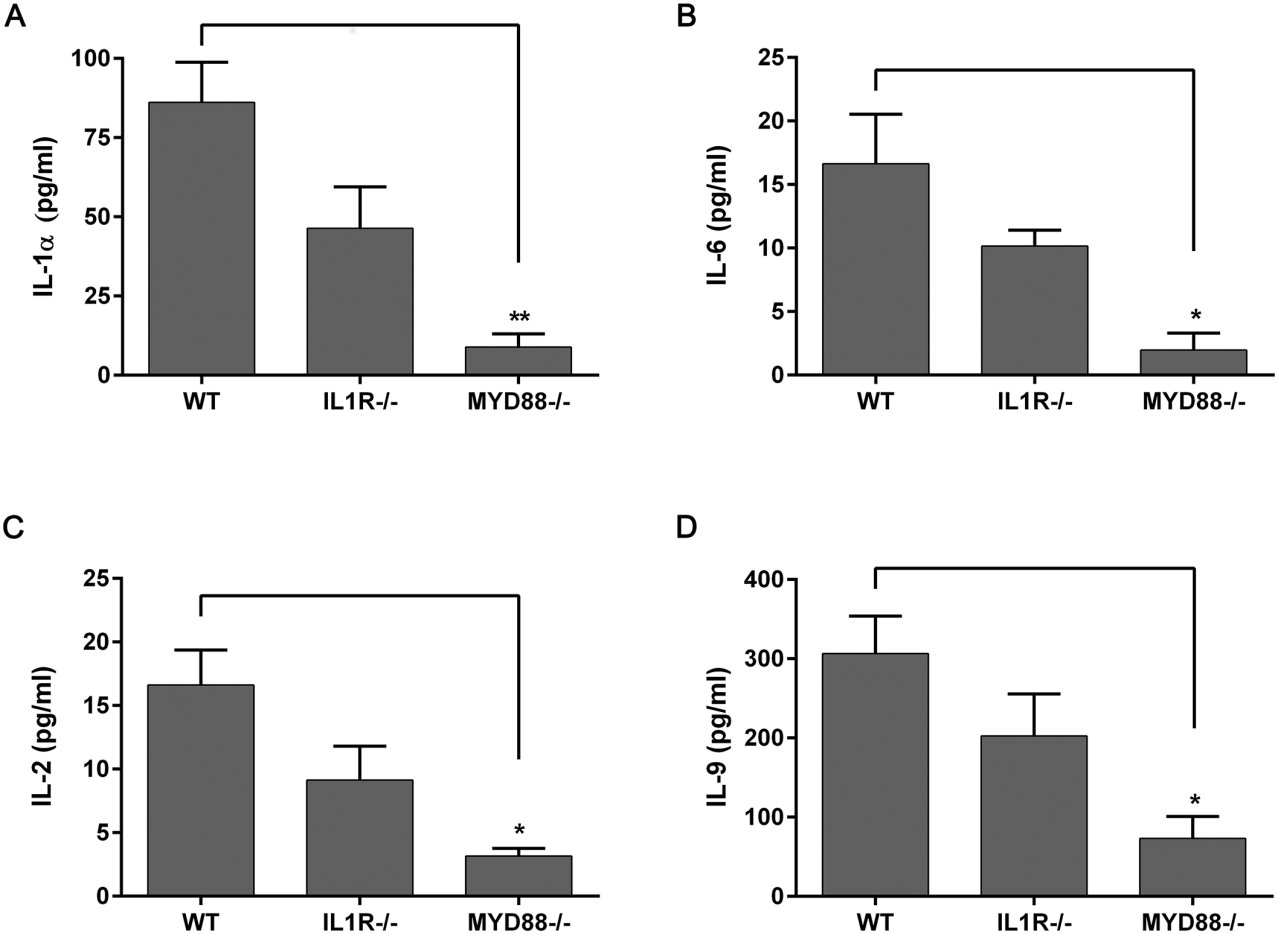
MyD88^-/-^ mice have decreased levels of cytokines in the conjunctiva. IL-1α (A), IL-6 (B), IL-2 (C), and IL-9 (D) expression were determined in untreated conjunctival homogenates from wild-type (WT), IL-1R^-/-^, and MyD88^-/-^ mice by Luminex multiplex assay. Data represent mean ± SEM of 3 independent experiments with each sample pooled from 5 mice. Analysis was performed by ANOVA with Bonferroni’s test for multiple comparisons, with comparison to WT samples. p<*0.05, **0.01.

Cytokine expression was also affected by lack of MyD88 signaling. In the untreated corneal epithelium, TNFα levels were significantly lower in MyD88^-/-^ mice, in both RNA expression (relative fold changes: WT, 2.46±0.75 compared to MyD88^-/-^, 0.16±0.02) (Fig 4B), as well as protein levels (WT, 2.04±0.12 pg/ml; MyD88^-/-^, 1.30±0.01 pg/ml) (Fig 4D). IL-1α expression was also significantly lower in MyD88^-/-^ animals, both in the cornea (WT, 25.4±6.1 pg/ml; MyD88^-/-^, 6.0±2.4 pg/ml) (Fig 4C) and conjunctiva (WT, 86.1±12.7 pg/ml; MyD88^-/-^, 8.9±4.1 pg/ml) (Fig 5A), as well as IL-6 (WT, 16.6±3.9 pg/ml; MyD88^-/-^, 1.96±1.3 pg/ml) (Fig 5B). Cytokines that are important for T lymphocyte activation, IL-2 and IL-9, were also reduced in the conjunctiva of MyD88^-/-^ animals (p<0.05) (Fig 5B, 5C and 5D).

### Corneal sensitivity is decreased in MyD88^-/-^ mice

**Fig 6:**
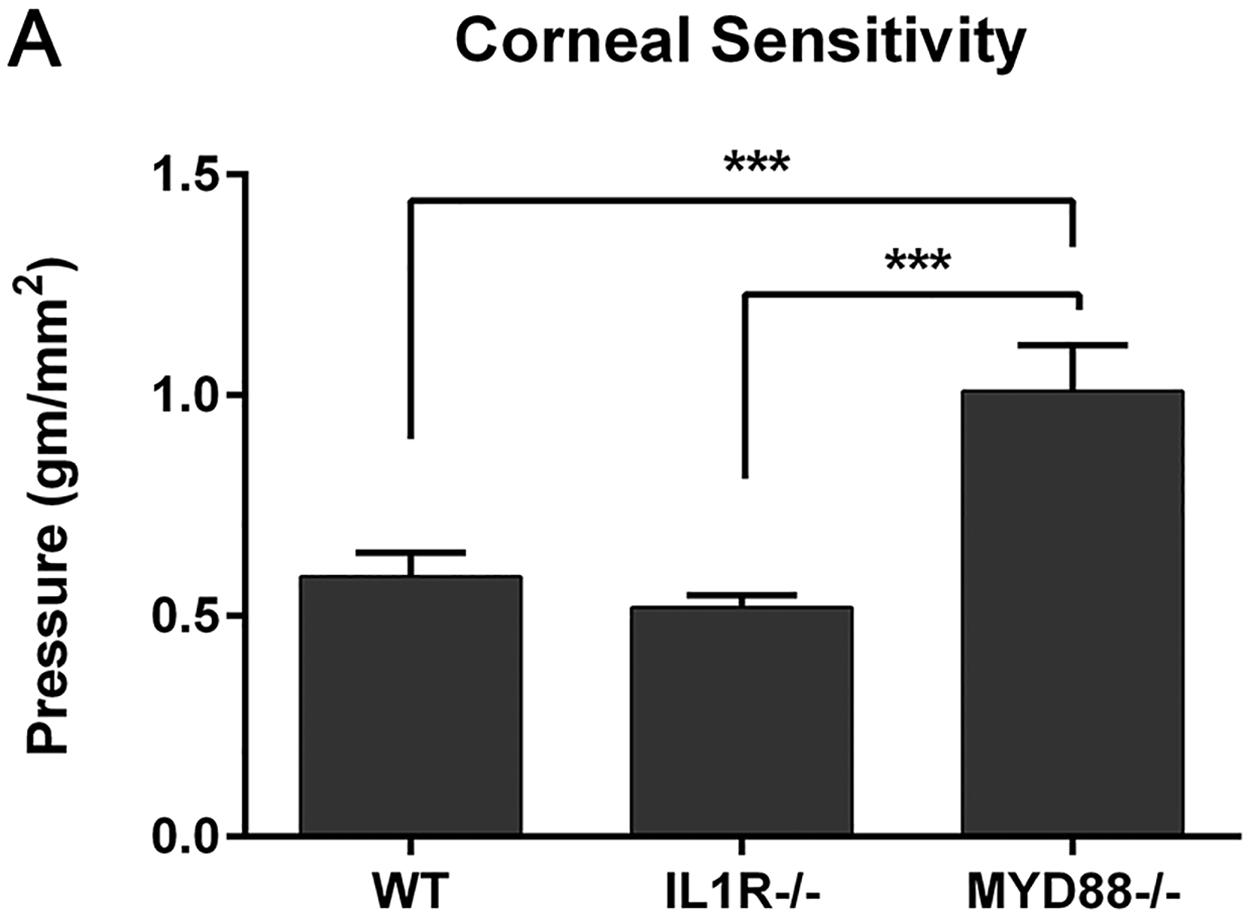
MyD88^-/-^ mice have decreased corneal sensitivity. Corneal sensitivity was measured in untreated mice using a Cochet-Bonnet esthesiometer, with greater pressure (gm/mm^2^) indicating lower sensitivity. Graph represents mean ± SEM of 9 mice per genotype. Analysis was performed by ANOVA with Bonferroni’s test for multiple comparisons. p<***0.001.

Inflammatory cytokines as well as TLRs have been reported to influence nerve function [27–31,39,40]With lower levels of cytokine expression and a lack of TLR signaling in the MyD88^-/-^ mice, we next sought to determine if corneal sensitivity was altered in these animals. A Cochet-Bonnet esthesiometer was applied to the central cornea to measure the threshold sensitivities in normal WT, IL-1R^-/-^, and MyD88^-/-^ mice. Interestingly, the amount of applied pressure needed to elicit a blink response was greater (indicating lower corneal sensitivity) in the MyD88^-/-^ mice (1.01±0.3 gm/mm^2^) compared to both WT (0.59±0.16 gm/mm^2^) and IL-1R^-/-^ (0.52±0.08 gm/mm2) (p<0.001, n = 9; Fig 6). This is a novel finding, implicating TLR signaling with maintenance of corneal sensitivity.

### MyD88 deficiency results in lower baseline expression of chemokines

**Fig 7:**
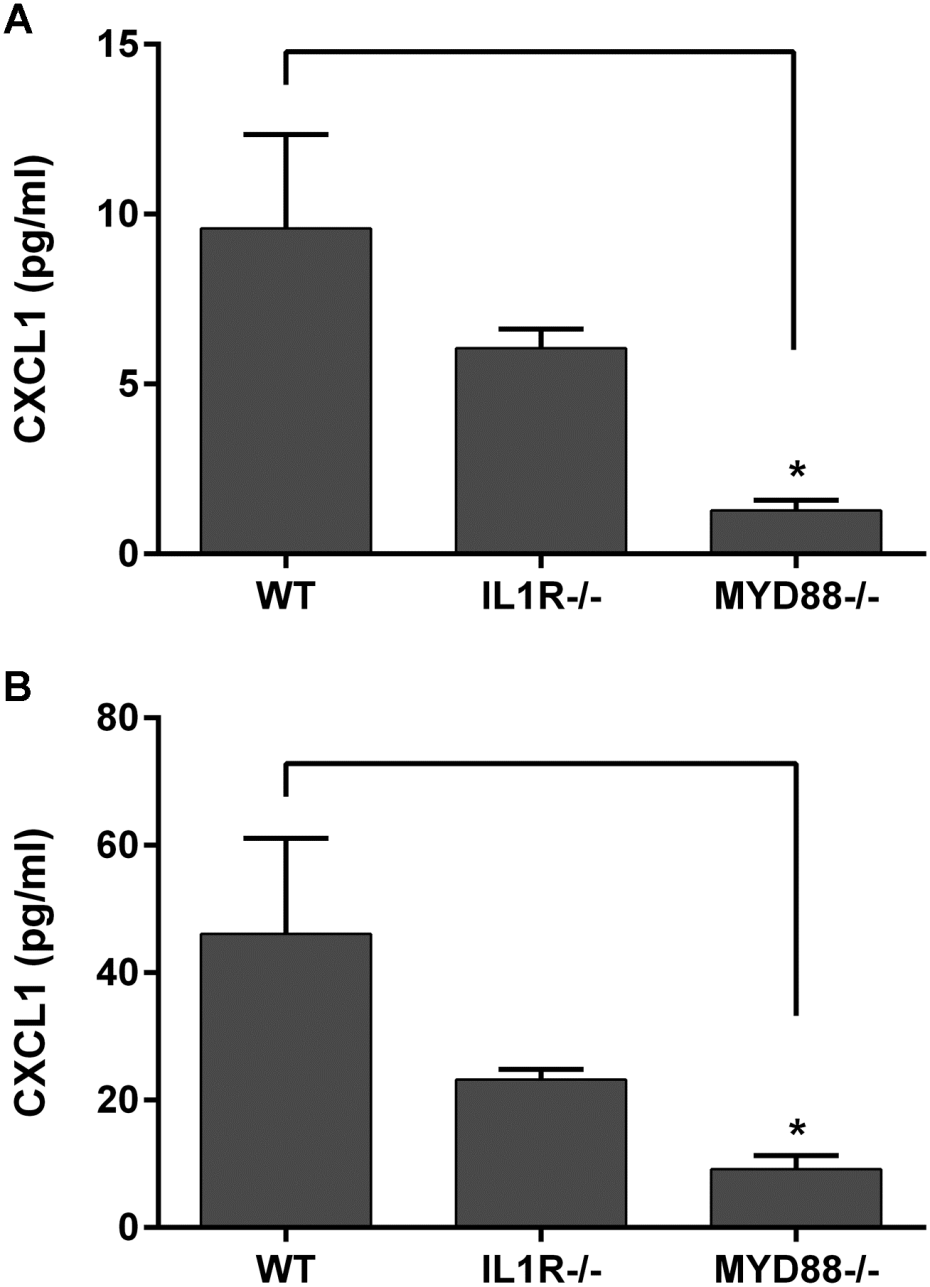
Chemokine CXCL1 is decreased in MyD88^-/-^ mice. CXCL1 chemokine expression was determined in untreated corneal (top) and conjunctival (bottom) lysates from WT (C57), IL-1R^-/-^, and MyD88^-/-^ mice by Luminex multiplex assay. Data represent mean ±SEM of 3 independent experiments with each sample pooled from 5 mice. Analysis was performed by ANOVA with Bonferroni’s test for multiple comparisons. p<*0.05.

Chemokines are important communicators that attract immune cells to sites of injury, infection, and inflammation. CXCL1 (C-X-C motif ligand 1) has been shown to be upregulated by TLR/MyD88 activation and is important in corneal wound healing and infection [18,41–43] Therefore, we examined the baseline expression of this chemokine in untreated mice deficient in MyD88 or IL-1R. CXCL1 protein levels were significantly decreased in the MyD88^-/-^ cornea (1.3±0.3 pg/ml vs. WT, 9.6±2.8 pg/ml) and conjunctiva (9.2±2.1 pg/ml vs. WT, 46.1±15 pg/ml) (Fig 7A and 7B). Although IL-R^-/-^ mice had lower mean protein concentrations in the cornea and conjunctiva, they were not significantly decreased from WT levels.

### MyD88^-/-^ mice have better clinical scores during PA infection but have greater numbers of isolated corneal bacteria

**Fig 8:**
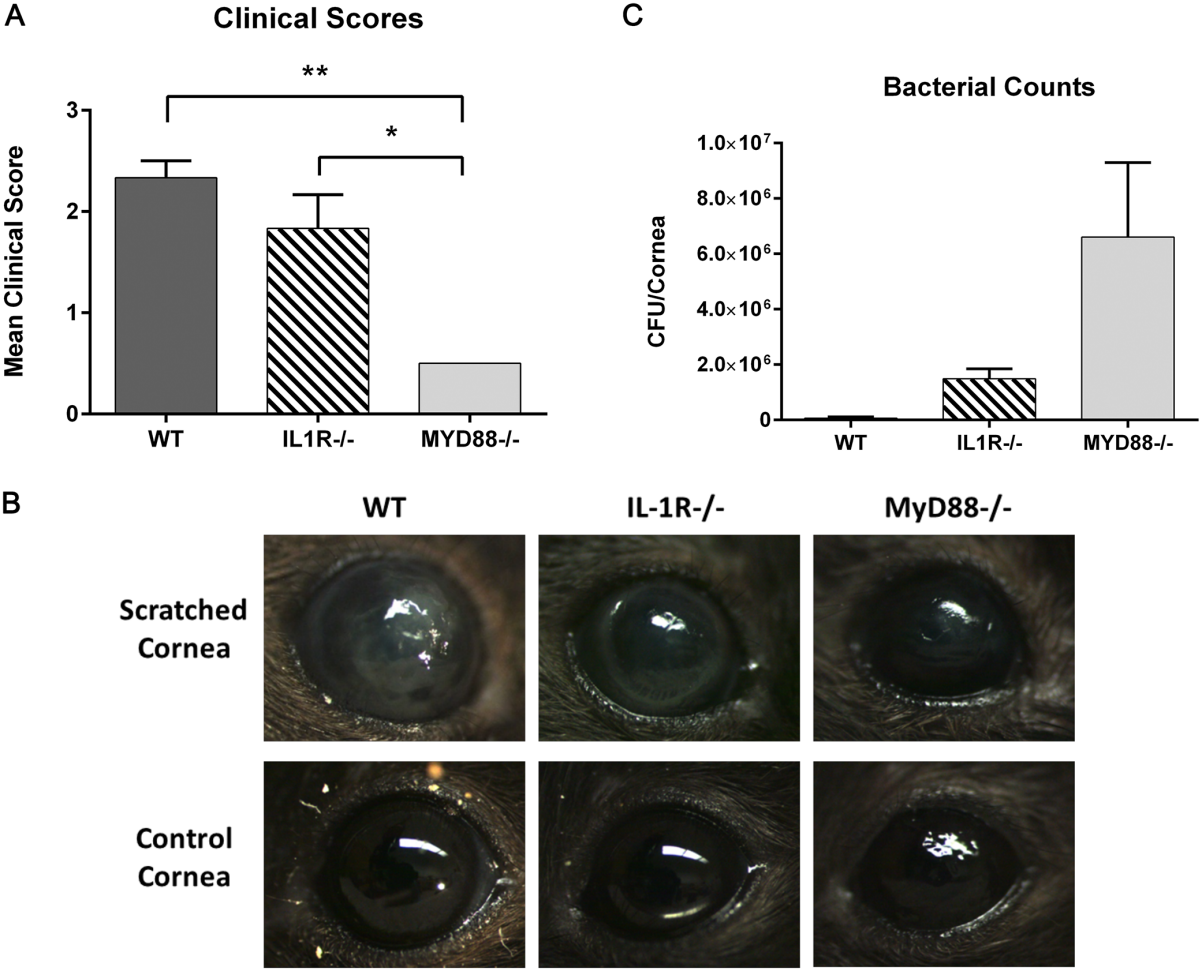
MyD88^-/-^ mice infected with PA were protected from inflammatory corneal infiltrates; however, more bacteria were isolated from MyD88^-/-^ corneas compared to both WT and IL-1R^-/-^. Corneas of WT (C57), IL-1R^-/-^, and MyD88^-/-^ mice were scratched and infected with 1.0 × 10^6^ CFU GFP-PA01. (A-B) After 24 hours, eyes were imaged by slit lamp microscopy and graded based on severity of infection. Images are representative of 3 animals per group. Analysis was performed by ANOVA with Bonferroni’s test for multiple comparisons. p<*0.05, **0.01 (B) For bacterial counts, corneas were harvested 24 hours post-infection, homogenates plated, and incubated for 16 hours at 37°C. The number of colonies was counted and data represent 3 corneas per group.

TLR activation and chemokine production are necessary for effective defense against corneal infection in response to PA challenge. Mice deficient in TLR signaling are not able to recruit neutrophils to the cornea, preventing clearance of the bacteria [18,44,45]. Similarly, IL-1R-deficient mice also have decreased immune cell infiltration and higher corneal bacterial counts compared to PA infected C57BL/6 WT [18]. Therefore, we wanted to directly compare the response of MyD88^-/-^ and IL-1R^-/-^ animals during pseudomonas keratitis, differentiating between the contributions of TLR and IL-1 signaling through MyD88, within the same experiment. WT, IL-1R^-/-^, and MyD88^-/-^ mice were inoculated with 10^6^ CFU GFP-PA01, following a corneal scratch wound, and clinical signs of infection evaluated by slit lamp examination after 24 hours. WT mice had dense corneal opacities, or cloudiness, which fully covered the pupil (2.25±0.12 clinical grading score) (Fig 8A and 8B). IL-1R^-/-^ animals consistently had a corneal ring infiltrate, a concentric opacity surrounding the cornea (1.5±0.3 clinical grading score). In contrast, MyD88^-/-^ corneas had very little to no corneal opacities, or no clinical signs of infection, indicating a lack of inflammatory cell infiltration (0.5±0.0 clinical grading score, p<0.01 compared to WT, p<0.05 compared to IL-1R^-/-^).

Although there was less clinical evidence of infection and corneal infiltrates, a greater number of viable bacteria were isolated from MyD88^-/-^ corneas (6.6 x 10^6^±2.7 x 10^6^ CFU/cornea), isolated 24 hours post-infection, compared to both IL-1R^-/-^ (1.5 x 10^6^±0.4 x 10^6^ CFU/cornea) and WT (0.06 x 10^6^±0.05 x 10^6^ CFU/cornea) (Fig 8C). This confirms that TLR-mediated MyD88 activation is necessary for effective clearance of bacteria from the ocular surface during pseudomonas keratitis.

Previous studies suggest that MyD88 is not only important for bacterial clearance, but also for bacterial adhesion and traversal into the epithelium [46–48]. MyD88-deficiency results in compromised barrier function and enhanced infection initiation. Therefore, we examined the effect of GFP-PA-01 inoculation on unscratched, intact corneas. At 6 and 24 hours post-challenge, MyD88 eyes did not show any signs of infection, regardless of strain, by slit lamp examination (data not shown). In addition, no viable bacteria were isolated or cultured from these corneas. We also examined corneal whole mounts to visualize bacterial penetration and infection. Scratched corneas corroborated cultured bacterial counts, with more GFP-labeled PA01 in MyD88^-/-^ corneas compared to WT (S1 Fig). However, no GFP-PA-01 were visible in unscratched MyD88^-/-^ corneas.

## Discussion

The cornea and conjunctiva form the external covering of the eye and are responsible for defending the rest of the eye from damage, protecting not only against physical injury, but microbial infection as well. In this study, we demonstrated that TLR stimulation increased MMP and cytokine expression in human corneal cells, initiating a protective inflammatory response to microbial ligand exposure. Further, baseline levels of inflammatory mediators were affected by lack of MyD88 and TLR signaling, as well as corneal sensitivity, *in vivo*, demonstrating the contribution of TLRs in defending the ocular surface from both pathogens and injury. The ocular surface is constantly exposed to microbes, commensal flora and opportunistic pathogens, as well as endogenous TLR ligands, or DAMPs. Baseline TLR activation provides a homeostatic level of MyD88 signaling, resulting in physiological levels of cytokine, chemokine, and MMP expression, as shown in this study. The baseline expression of inflammatory mediators may aid in defending the ocular surface from pathogenic challenge and injury, enhancing the state of readiness of the tissue to respond rapidly to insult. This would explain, in part, the increased susceptibility to injury and infection seen with MyD88 deficiency, not only in the cornea, as we demonstrate with pseudomonas keratitis, but in other tissues as well [13,14,18,20,49,50].

In particular, epithelial cells play a vital role in maintaining ocular surface homeostasis and regulating interactions with immune cells, as well as underlying stromal cells, during injury and infection. Epithelial crosstalk serves to enhance protection, aid in wound healing, and maintain barrier function [51,52]. An important aspect of this epithelial communication is mediated through TLR signaling. Others have reported that the interaction between commensal microbes and epithelial cells, through TLR recognition, plays an integral role in maintaining tissue homeostasis [49]. In the lung, for example, TLR2 and TLR5 ligands stimulate bronchial epithelial cell growth and survival, offer protection from apoptosis, and maintain epithelial integrity [53]. In the intestine, TLR recognition of commensal microflora plays an important role in propagating epithelial homeostasis and protection from colonic injury [49]. Interestingly, in corneal epithelial cells, TLR5 activation, through flagellin exposure, confers protection against infection development, increasing the innate protective function of the corneal epithelium [17,54]. It is important to note that the animals used in the current study were housed in a minimal barrier facility and not in a germ-free environment. Therefore, the baseline expression of cytokines and chemokines in ocular surface tissue was likely a result of interaction and stimulation of epithelial cells by commensal microbes. Dissecting the role of ocular surface microbiota in protecting against infection, enhancing barrier function, and augmenting the innate immune system would be extremely interesting to study further.

One mechanism for this protective TLR function was demonstrated in the intestine to be the MyD88-dependent production of baseline levels of cytokines and chemokines, such as IL-6, TNFα, and CXCL1 [49]. These mediators have been shown to be beneficial in protecting multiple cell types from injury, and are present under normal, steady-state conditions, enhancing the ability of cells to respond to injury and infection [55–58]Studies in various cell types demonstrate that in addition to stimulating inflammation, these factors are protective, and are expressed at baseline levels in non-pathological conditions. In the normal cornea and conjunctiva, for example, MMP-9 is present and is upregulated during wound healing and inflammation, enhancing epithelial turnover and degrading adhesion complexes on cell membranes and extracellular matrix proteins [25,59,60]. IL-1 is also present and its expression is critical for initiation of wound healing and communication between the corneal epithelium and stroma [24,61]. Cytokines such as IL-1β and TNFα have also been shown to increase the expression of antimicrobial peptides, aiding in the defense of the ocular surface against pathogens [62]. IL-1β is necessary to induce chemokine expression, early in PA corneal infection, for recruitment of leukocytes to the site of infection [18,41,63,64]. CXCL1 mediates effective clearance and resolution of corneal infections, and is critical for pseudomonas keratitis, in particular [18,41]. This chemokine is also required for efficient corneal wound healing, recruiting neutrophils to the ocular surface, and also functions to induce proliferation of some cell types [65–67]Indeed in the current study, we have shown that TLR-dependent MyD88 signaling is necessary for baseline expression of cytokine and chemokine levels and effective defense against bacterial challenge.

TLR activation is known to increase expression of inflammatory cytokines as well as MMPs at the ocular surface, which aid in the immune response to infection and injury. In *in vitro* studies, we demonstrated that primary corneal and conjunctival epithelial cells respond to TLR agonist treatment, resulting in upregulation of MMPs, IL-6, and IL-8. Treatment with FSL-1, a lipoprotein that binds to TLR2/1, and flagellin, a bacterial protein that activates TLR5, elicited the strongest response in increasing these inflammatory mediators. During corneal infections, IL-1β, IL-6, IL-8, and TNFα are important in the initiation of inflammatory signals [68] and both TLR2 and TLR5 mediate microbial clearance and cytokine production during *S*. *aureus* and PA keratitis [6,11,14,17,18,69]. Therefore, our data support *in vivo* studies demonstrating TLR involvement in infection, and show that ocular surface epithelial cells mediate cytokine production upon stimulation, providing necessary signals for an effective response.

MyD88 is an essential adaptor molecule required for both TLR and IL-1R signaling [3,7,8]. Given that MyD88 is involved in both TLR and IL-1R signaling, IL-1R-deficient mice were also included in this study, in order to differentiate the effects between TLR and IL-1R signaling. Although IL-R^-/-^ mice had lower average cytokine and chemokine concentrations in the cornea and conjunctiva compared to WT, they were not significantly decreased, thus demonstrating that TLR pathways are responsible, in part, for the decreased cytokine and expression seen in the MyD88^-/-^ animals.

MyD88 has been shown to be important in protecting the corneal epithelium from PA adhesion and further, in preventing bacterial traversal [46]. Therefore, MyD88 confers protection not only in initiating an immune response to pathogenic challenge, but also in stopping bacterial entry and penetration into the corneal epithelium [48]. For this reason, we were interested in evaluating visible clinical manifestations of infection as well as corneal bacterial load early in the course of infection, directly comparing these parameters in MyD88- and IL-1R-deficient animals. In our scratch infection model, following 24 hours of PA challenge, MyD88^-/-^ mice had very little evidence of corneal infiltrates, or no clinical signs of infection, indicating a lack of inflammatory cell recruitment. This is consistent with the data demonstrating that MyD88/TLR signaling is necessary for CXCL1 expression, which attracts neutrophils to the site of infection necessary for bacterial clearance. In contrast, both IL-1R^-/-^ and WT mice had dense corneal opacities, or cloudiness, indicating neutrophil infiltration. In line with these observations, MyD88-deficient mice had a greater number of viable bacteria isolated from their corneas, compared to both IL-1R^-/-^ and WT. These results demonstrate that TLR-mediated MyD88 activation is necessary for effective clearance of bacteria from the ocular surface during pseudomonas keratitis, as well as leukocyte infiltration, and chemokine expression, furthering the concept that TLR and MyD88 are protective at the ocular surface. While Sun et al. showed similar results, showing increased bacterial load and decreased clinical scores in knockout animals, evaluation of MyD88^-/-^ and IL-1R^-/-^ mice in the same experiment, allow direct comparison between their responses to infection, demonstrating the specific importance of TLR signaling in corneal defense during bacterial challenge.

The ocular surface is very good at preventing and protecting the eye from infection. Despite constant bombardment and exposure to pathogenic challenge, healthy corneas resist infection. In animal models of bacterial keratitis, inoculating the ocular surface with large concentrations of topical PA does not illicit an infection. Corneas must be compromised by an injury, such as a scratch or blotting, to allow bacterial adhesion and subsequent epithelial traversal and infection. Mice with deficiencies in corneal barrier function, or genes that are important for corneal defense, have compromised ability to resist infection. Tam et al. (2011) showed that PA-01 were able to adhere and penetrate into the corneal epithelium of MyD88^-/-^ mice without prior injury, unlike control C57 animals [46]. In the current study, we sought to visualize and isolate bacteria from uninjured MyD88^-/-^ corneas, in order to quantify penetrating bacteria without a scratch injury and evaluate clinical signs of infection in the knockout animals. However, in our model, we were not able to see any evidence of infection, culture PA01, or visualize adhering bacteria using fluorescent microscopy, without a corneal scratch. One reason for this is likely the lower amount of bacterial inoculum used in our study (10^6^ CFU). Also, small amounts of bacterial adhesion and traversal might not result in corneal infection. Compensatory mechanisms, or resident immune cells, might be sufficient to eliminate bacteria and prevent infection from developing. Therefore, it is not surprising that we were not able to culture bacteria from the uninjured corneas. We have previously shown that TLR activation increases antimicrobial peptide expression [4,5]; however, interestingly, we did not see a change in either CRAMP or mBD3 in the corneas of MyD88^-/-^ mice.

The cornea is a highly innervated tissue, which serves to protect the eye from damage, as well as providing trophic factors that maintain corneal health [70–73] In other tissues, TLRs, MyD88 signaling, and inflammatory cytokines have been implicated in nerve function and induction of chronic pain [27–30]. With lower levels of cytokine expression and a lack of TLR signaling in the MyD88^-/-^ mice, our hypothesis was that corneal sensitivity would be affected in the knockout animals. Interestingly, our data supported this hypothesis. Compared to WT and IL1R^-/-^, MyD88^-/-^ mice had lower corneal sensitivities, as measured by anaesthesiometry. Lower sensitivity to mechanical stimuli leaves the cornea more susceptible to damage, increasing the amount of stimulus needed to elicit a protective blink. Therefore, these results demonstrate that TLR-MyD88 signaling is protective, in maintaining a healthy corneal sensitivity. This finding is novel and demonstrates the need for further exploration into the role of TLRs in corneal nerve function.

Although signaling must be tightly regulated and controlled to prevent tissue damage, chronic inflammation, and maintain corneal transparency, TLR-MyD88 signaling is important for the cytoprotective expression of cytokines and chemokines, protection against corneal infection, and maintenance of corneal sensitivity. This study provides evidence that baseline TLR signaling is beneficial to the ocular surface, which should be considered when blocking TLRs for therapeutic purposes. This work also furthers our understanding of the importance of TLR signaling in corneal defense and immune homeostasis, showing that a lack of MyD88 may compromise the baseline innate response to insult.

## Supporting information

S1 Fig GFP-labeled PA-01 in corneal whole mounts. Corneas of WT (C57) and MyD88^-/-^ mice were scratched and inoculated with 1.0 × 10^6^ CFU GFP-PA01. After 24 hours, eyes were excised and corneas mounted for whole mount imaging. Image is a representative image of the central corneal region, showing GFP (green) fluorescing PA01. Scale bar = 15μm.
(TIF)
