# Supplementary figures and images for: MyD88 contribution to ocular surface homeostasis
Source: PLoS One. 2017 Aug 10;12(8):e0182153. doi: 10.1371/journal.pone.0182153 (PMC5552092; doi:10.1371/journal.pone.0182153)

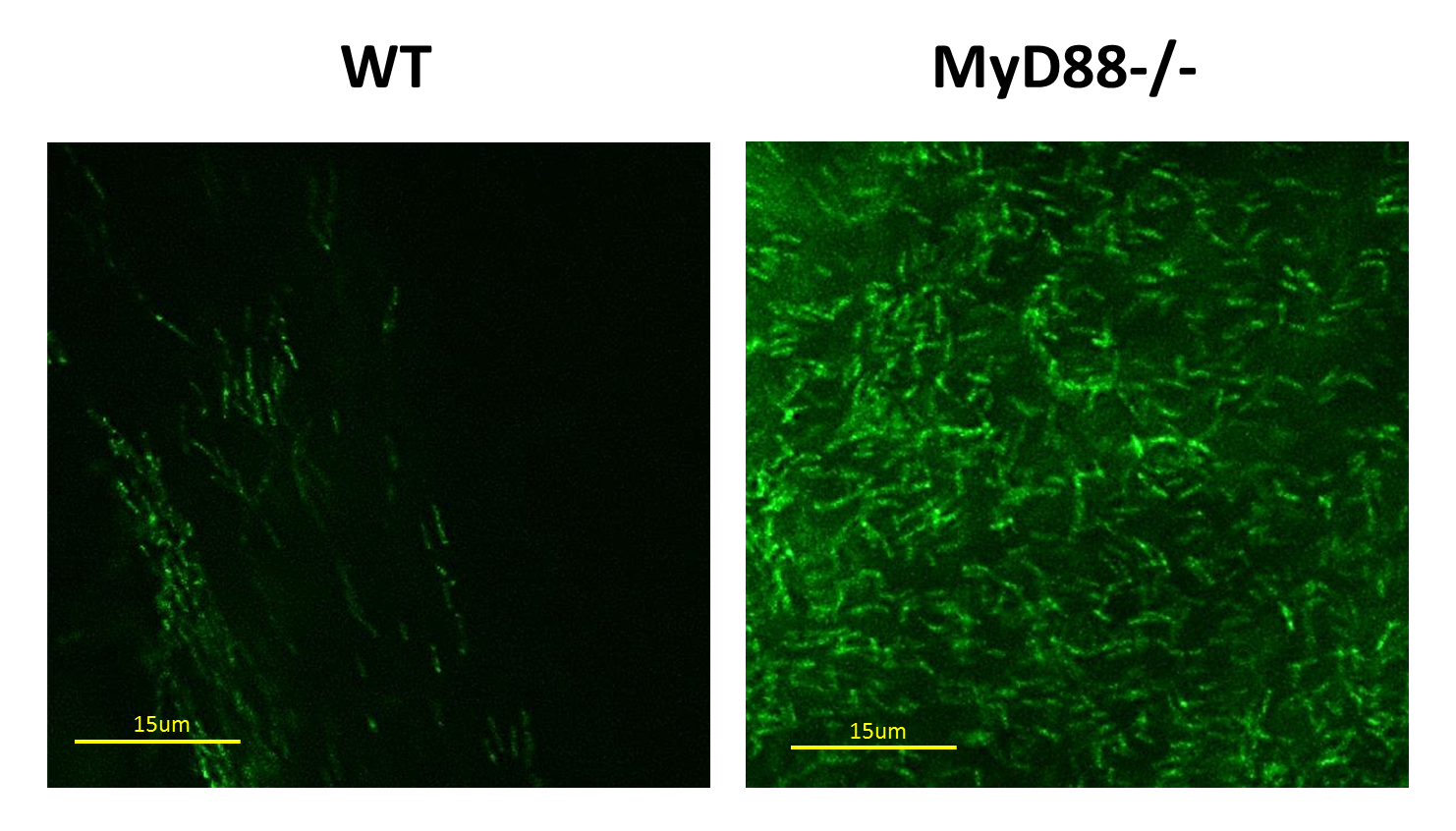

Supplement: S1 Fig — Corneas of WT (C57) and MyD88-/- mice were scratched and inoculated with 1.0 × 10^6^ CFU GFP-PA01. After 24 hours, eyes were excised and corneas mounted for whole mount imaging. Image is a representative image of the central corneal region, showing GFP (green) fluorescing PA01. Scale bar = 15μm. (TIF) [file pone.0182153.s001.tif]
